# Physical Activity–Friendly Policies and Community Design Features in the US, 2014 and 2021

**DOI:** 10.5888/pcd20.220397

**Published:** 2023-08-17

**Authors:** Bryant J. Webber, Geoffrey P. Whitfield, Latetia V. Moore, Ellen Stowe, John D. Omura, Anu Pejavara, Deborah A. Galuska, Janet E. Fulton

**Affiliations:** 1 Division of Nutrition, Physical Activity, and Obesity, National Center for Chronic Disease Prevention and Health Promotion, Centers for Disease Control and Prevention, Atlanta, Georgia; 2 Epidemic Intelligence Service, Centers for Disease Control and Prevention, Atlanta, Georgia; 3 Oak Ridge Institute for Science and Education Research Participation Program, Oak Ridge, Tennessee; 4 Division of Population Health, National Center for Chronic Disease Prevention and Health Promotion, Centers for Disease Control and Prevention, Atlanta, Georgia

## Abstract

**Introduction:**

The 2014 Community-Based Survey of Supports for Healthy Eating and Active Living documented the prevalence of US municipal policy and community design supports for physical activity. The survey was repeated in 2021. Our study examined change in the prevalence of supports from 2014 to 2021, overall and by municipality characteristic.

**Methods:**

Municipalities were sampled independently each survey year. We calculated prevalence in 2014 and 2021 and the prevalence ratio (PR) for 15 supports covering zoning codes, park policies and budgets, design standards, Complete Streets policies, and shared use agreements. We used a Bonferroni-corrected Breslow-Day test to test for interaction by municipality characteristic.

**Results:**

In 2014 (2,009 municipalities) compared with 2021 (1,882 municipalities), prevalence increased for several zoning codes: block sizes of walkable distances (PR = 1.46), minimum sidewalk width (PR = 1.19), pedestrian amenities along streets (PR = 1.15), continuous sidewalk coverage (PR = 1.14), and building orientation to pedestrian scale (PR = 1.08). Prevalence also increased for design standards requiring dedicated bicycle infrastructure for roadway expansion projects or street retrofits (PR = 1.19). Prevalence declined for shared use agreements (PR = 0.87). The prevalence gap widened between the most and least populous municipalities for Complete Streets policies (from a gap of 33.6 percentage points [PP] in 2014 to 54.0 PP in 2021) and for zoning codes requiring block sizes that were walkable distances (from 11.8 PP to 41.4 PP).

**Conclusion:**

To continue progress, more communities could consider adopting physical activity–friendly policies and design features.

**Summary**

**What is already known?**

Physical activity is influenced by community-level factors. A 2014 survey of US municipalities determined the prevalence of policy and community design supports for physical activity–friendly places.

**What is added by this report?**

Prevalence of some municipal supports for physical activity was higher in 2021 than in 2014. Adoption of Complete Streets policies and zoning codes for block size disproportionately increased in more populous municipalities. Adoption of zoning codes for mixed land use disproportionately increased in municipalities where most of the population had at least some college education.

**What are the implications for public health practice?**

Additional communities could consider adopting physical activity–friendly policies and design features.

## Introduction

Being physically active is one of the best ways to improve and preserve health. Regular physical activity enhances daily quality of life and reduces the incidence or severity of many diseases (1). However, physical activity is often influenced by contextual factors that lie beyond a person’s control. Community-level strategies to increase physical activity may reach a larger audience than those directed at the individual (2).

Community design approaches offer an evidence-based solution to increasing participation in physical activity by making it safer and more accessible to everyone (3). Strategies range from updating zoning codes that improve overarching land use patterns to tangible changes in the built environment, such as adding street furnishings (eg, benches) and park lighting. Effective strategies include pedestrian and bicycle network expansion through well-maintained sidewalks and bicycle lanes, initiatives such as Safe Routes to School, and policies such as Complete Streets (4). The last is an infrastructure approach that considers the ages, abilities, and transportation modes of all users (including pedestrians and bicyclists) as part of major road project designs, thereby enhancing safety and opportunities for active transportation (4).

To better understand the uptake of municipal-level supports for physical activity, in 2014 the Centers for Disease Control and Prevention (CDC) launched the Community-Based Survey of Supports for Healthy Eating and Active Living (CBS HEAL) (5). This nationally representative survey of municipalities documented the prevalence of policy and community design supports for physical activity and identified differences by geographic and sociodemographic characteristics (6–9). Communities with smaller and less formally educated populations and those located in the South were less likely to report many policy and community design supports (6–8). Complete Streets policies, for example, were twice as common among communities in the West as in the South, adjusted for population size, urbanicity, and socioeconomic status (7). Some interventions, such as budget provisions for park maintenance and lighting, were less common in communities with fewer than 2,500 people or with a lower percentage of college-educated residents (8).

Ongoing surveillance of changes in the presence of these community-level supports is important for identifying where improvements are occurring, where inequitable distribution is widening or narrowing, and where resources may need to be prioritized. To obtain updated information, CDC administered CBS HEAL again in 2021. The objective of our study was to examine changes in prevalence of US municipal policy and community design supports for physical activity from 2014 to 2021, and to determine if any changes differed by municipality characteristic.

## Methods

### Community-Based Survey of Supports for Healthy Eating and Active Living (CBS HEAL) overview

CBS HEAL is a nationally representative survey of US municipalities that collects information on environmental and policy supports for healthful diets and regular physical activity. The survey is administered by CDC’s Division of Nutrition, Physical Activity, and Obesity. Our study used cross-sectional data from the 2014 and 2021 CBS HEAL surveys. Detailed methodology of the 2014 survey is available elsewhere (6). Responses for the second administration of CBS HEAL were collected from May through September 2021. As with the 2014 survey, all US municipalities with a population of at least 1,000 people (N = 10,300 municipalities) were eligible for selection; population estimates were derived from the 2017 Census of Governments file (10). To achieve a nationally representative sample, municipalities were randomly selected after stratification by census region (Northeast, Midwest, South, or West) and by urbanicity status (urban or rural). To define urbanicity, the percentage of each municipality’s population that lived in a census-designated urban area was obtained from the 2010 US Census Urban Area to Place Relationship file. Municipalities with percentages above the 30th percentile of the national distribution were classified as urban. Based on the sampling frame, a total of 4,417 municipalities were invited to participate (11).

### Questionnaire contents

We investigated 15 questions related to policy and community design supports for physical activity that were included in CBS HEAL for both 2014 and 2021. These questions fall into 4 categories: zoning codes design/development guidelines (6 questions); policies or budget provisions related to parks or outdoor recreation areas (4 questions); design standards, guidelines, and policies (3 questions); and other supports (2 questions) (Table 1). We excluded municipalities missing a response on at least 1 of these 15 questions.

**Table 1:** Analysis of Physical Activity Support Questions, Community-Based Survey of Supports for Healthy Eating and Active Living (CBS HEAL), 2014 and 2021^a^

Feature	Question^a^
**Zoning codes design and development guidelines**	
Variable	Does your local government include the following features in policies for development, including zoning codes design/development guidelines that
Block size	Require short to medium pedestrian-scale block sizes?
Continuous sidewalks	Require continuous sidewalk coverage?
Sidewalk width	Require minimum sidewalk widths of 5 feet?
Building orientation	Require buildings to be oriented to pedestrian scale (eg, entrances or windows face the street, reduced front setbacks)?
Pedestrian amenities	Require pedestrian amenities such as trees or furniture along the street?
Allow mixed land uses	Allow mixed land uses (eg, zoning that combines residential land use with one or more commercial, institutional, or public land uses)?
**Policies or budget provisions related to parks or outdoor recreation areas**	
Variable	Does your local government have policies or budget provisions related to parks or outdoor recreation areas, such as
Lighting	Lighting in parks or outdoor recreation areas?
Patrols	Patrols by police or security in parks or outdoor recreation areas?
Maintenance	Maintenance of green space and equipment?
Dog leashes	Prohibition of unleashed or unrestrained dogs in parks and outdoor recreation areas (excluding dog parks)?
**Design standards, guidelines, and policies**	
Variable	Does your local government have design standards, guidelines, or policies that require
Roadway expansion	Installation of dedicated bicycle infrastructure for roadway expansion projects or when retrofitting streets?
Bicycle space reservation	Developers to reserve space for use by the local jurisdiction for development of bicycle infrastructure?
Traffic-calming features	Traffic-calming features (eg, speed bumps, reduced speed zones, signal modifications) that increase roadway safety in areas with high pedestrian and bicycle volume (not including school zones)?
**Other supports**	
Complete Streets policy	Does your local government have a formal Complete Streets policy, as defined by the National Complete Streets Coalition, for designing and operating streets with safe access for all users?
Shared use agreement	Has your local government adopted a joint or shared use agreement or memorandum of understanding with any school that allows the public to use school recreational facilities (eg, gymnasiums, athletic fields, playgrounds) during nonschool hours?

a Centers for Disease Control and Prevention. Community-based survey of supports for healthy eating and active living (5). Response options were “yes,” “no,” or “don’t know”; shared use agreement also included the option of “our municipality does not have schools in our jurisdiction.”

### Questionnaire administration

For each sampled municipality, the web-based questionnaire was sent to a city or town planner or someone with an equivalent title. The primary respondent could electronically nominate someone in the municipality to complete a particular questionnaire section to enhance completion and accuracy. For instance, the primary respondent could refer park questions to the director of the parks and recreation department. (Although referral to municipal experts was encouraged in 2014, the digitized nominate feature was not added until 2021.) Respondents could select “yes,” “no,” or “don’t know,” or they could leave the answer blank. We defined a “don’t know” response as “no” for all primary analyses, and blank answers were excluded.

### Municipality characteristics

We used data from the US Census Bureau, merged by Federal Information Processing Standards (FIPS) place codes, to characterize municipalities by population size, urbanicity, region, race or ethnicity, education, and poverty. We stratified population size as small (1,000–2,499 people), medium (2,500–49,999 people), or large (≥50,000 people) by using the 2007 and 2017 Census of Governments files (10) for the 2014 and 2021 surveys, respectively. Urbanicity was defined differently for the analyses than for the sampling plan (11). For analyses, we dichotomized urbanicity as either urban or rural, with urban defined as having more than 50% of the population living in an urban area, according to population data from the 2012 and 2017 Census of Governments files and land area from the US Census Urban Area to Place Relationship files (12). Regions, based on US Census Bureau schema, were classified as Northeast, Midwest, South, or West (13).

Sociodemographic categories for the respective surveys were based on 5-year population estimates from the 2013 and 2020 American Community Survey (14). We categorized race and ethnicity as majority (>50%) or minority (≤50%) non-Hispanic White. For education we restricted the population to residents aged 25 years or older and dichotomized as high school graduate or less (if the majority of municipality residents had only a high school diploma or less) and some college or more (if the majority had at least some college education). We categorized poverty as high if 20% or more of the population, or low if less than 20%, lived below the poverty threshold at the time of the survey, defined by the total family income in the last 12 months, family size, and household composition (15).

### Statistical analysis

We compared municipality characteristics between the 2014 and 2021 CBS HEAL by using the Wald χ^2^ test. For each of the physical activity supports, we calculated prevalence in 2014 and 2021 with 95% CIs. We also calculated the unadjusted prevalence difference (PD, defined as 2021 minus 2014) and the unadjusted prevalence ratio (PR, defined as 2021 divided by 2014). CIs were estimated via the Taylor linearization method, and significance was established at a 2-sided *P* < .05. We used the Breslow–Day test to determine if any municipality characteristic modified the prevalence change between 2014 and 2021, defining significance as a Bonferroni-corrected *P* < .008. For significant associations, we developed slope graphs to depict the change by the effect-modifying characteristic.

With the exception of these effect-modifying associations, we investigated measured confounding by municipality characteristic by using Mantel-Haenszel tests and multiple logistic regression. Because all adjusted PRs were within 10% of unadjusted estimates, we assumed that municipality characteristics were not substantively confounding the relationship between support prevalence and survey year. Subsequently, to simplify presentation of findings, we reported unadjusted PRs. Some municipalities participated in both the 2014 and 2021 surveys and were not independent; we performed a sensitivity analysis that excluded those municipalities. We also conducted a sensitivity analysis that excluded “don’t know” responses. We conducted all analyses in SAS version 9.4 (SAS Institute) and SAS-callable SUDAAN, release 11.0.0 (RTI International). Analyses accounted for the survey design, nonresponse, and weights.

## Results

### Municipality characteristics

Of the 2,029 municipalities that returned the questionnaire in 2014, the sample size was 2,009 after excluding those with missing answers; of the 1,982 who returned it in 2021, the sample size was 1,882 municipalities. Most municipalities in 2014 and 2021 were medium sized, urban, and located in the Midwest or South (Table 2). The proportion of municipalities with majority non-White populations increased from 13.4% in 2014 to 16.4% in 2021. In addition, the proportion of communities with most of the population having some college education increased from 55.5% to 67.7%; the proportion with a low poverty level also increased from 69.6% to 78.7%. Distributions of municipalities by population size, urban status, and region were similar in 2014 and 2021.

**Table 2:** Characteristics of US Municipalities, Community-Based Survey of Supports for Healthy Eating and Active Living, 2014 and 2021^a^

Characteristic	2014	2021	*P* value^b^
	N (weighted %)	N (weighted %)	
All	2,009 (100.0)	1,882 (100.0)	NA
**Population, no.**			
1,000–2,499	717 (35.0)	641 (33.9)	.80
2,500–49,999	1,151 (58.2)	1,093 (59.1)	
≥50,000	141 (6.9)	148 (7.0)	
**Urban status**			
Urban^c^	1,372 (69.9)	1,280 (70.0)	.97
Rural	637 (30.1)	602 (30.0)	
**Census region**			
Northeast	232 (14.5)	278 (13.7)	.92
Midwest	742 (35.1)	631 (35.2)	
South	703 (36.1)	540 (36.3)	
West	332 (14.3)	433 (14.7)	
**Racial or ethnic composition**			
>50% non-Hispanic White	1,742 (86.6)	1,578 (83.6)	.009
≤50% non-Hispanic White	267 (13.4)	304 (16.4)	
**Educational attainment**			
≤High school graduate	888 (44.5)	569 (32.3)	<.001
≥Some college	1,121 (55.5)	1,313 (67.7)	
**Poverty prevalence**			
High (≥20%)	610 (30.4)	380 (21.3)	<.001
Low (<20%)	1,399 (69.6)	1,502 (78.7)	

Abbreviation: NA, not applicable.

a Centers for Disease Control and Prevention. Community-based survey of supports for healthy eating and active living (5). Based on municipalities that responded “yes,” “no,” or “don’t know” for all 15 policy and community design supports.

b Based on the Wald χ^2^ test.

c Defined as more than 50% of the population residing within a census-designated urban area.

### Overall prevalence

The 2014 prevalence of supports ranged from 14.2% for block size zoning codes to 87.3% for park dog leash policies. In 2021, supports ranged from 20.9% for block size zoning codes to 86.0% for park maintenance policies. Of the 15 supports, prevalence significantly increased for 7, significantly decreased for 3, and remained statistically equivalent for 5 (Table 3).

**Table 3:** Prevalence, Prevalence Differences, and Prevalence Ratios of Policies and Community Design Supports for Physical Activity Among US Municipalities, Community-Based Survey of Supports for Healthy Eating and Active Living, US, 2014 and 2021^a^

Variable	2014 (n = 2,009)	2021 (n = 1,882)	Prevalence difference (95% CI)^c^	Prevalence ratio (95% CI)^d^
	Prevalence (95% CI)^b^	Prevalence (95% CI)^b^		
**Zoning codes design and development guidelines**				
Block size	14.2 (12.8 to 15.8)	20.9 (19.1 to 22.8)	6.6 (4.2 to 9.0)	1.46 (1.27 to 1.69)
Continuous sidewalks	40.1 (38.0 to 42.3)	45.7 (43.4 to 48.0)	5.5 (2.4 to 8.7)	1.14 (1.06 to 1.23)
Sidewalk width	43.9 (41.8 to 46.1)	52.2 (49.9 to 54.5)	8.3 (5.1 to 11.5)	1.19 (1.11 to 1.27)
Building orientation	38.7 (36.6 to 40.8)	42.0 (39.7 to 44.3)	3.3 (0.1 to 6.4)	1.08 (1.00 to 1.17)
Pedestrian amenities	33.6 (31.6 to 35.7)	38.6 (36.4 to 40.9)	5.0 (1.9 to 8.1)	1.15 (1.05 to 1.25)
Allow mixed land uses	69.0 (67.0 to 71.0)	70.2 (68.0 to 72.3)	1.2 (–1.8 to 4.1)	1.02 (0.97 to 1.06)
**Policies or budget provisions related to parks or outdoor recreation areas**				
Lighting	78.1 (76.2 to 79.9)	76.4 (74.4 to 78.4)	–1.7 (–4.4 to 1.0)	0.98 (0.95 to 1.01)
Patrols	84.6 (83.0 to 86.1)	75.6 (73.5 to 77.6)	–9.0 (–11.5 to –6.4)	0.89 (0.87 to 0.92)
Maintenance	86.9 (85.3 to 88.3)	86.0 (84.2 to 87.5)	–0.9 (–3.1 to 1.3)	0.99 (0.96 to 1.02)
Dog leashes	87.3 (85.8 to 88.7)	84.0 (82.2 to 85.7)	–3.3 (–5.6 to –1.0)	0.96 (0.94 to 0.99)
**Design standards, guidelines, and policies**				
Roadway expansion	27.2 (25.4 to 29.2)	32.4 (30.4 to 34.6)	5.2 (2.3 to 8.1)	1.19 (1.08 to 1.31)
Bicycle space reservation	18.7 (17.1 to 20.5)	22.6 (20.7 to 24.5)	3.8 (1.3 to 6.4)	1.21 (1.06 to 1.36)
Traffic-calming features	49.5 (47.3 to 51.7)	51.3 (49.0 to 53.6)	1.8 (1.4 to 5.0)	1.04 (0.97 to 1.10)
**Other supports**				
Complete Streets policy	25.0 (23.2 to 27.0)	25.9 (23.9 to 27.9)	0.9 (–1.9 to 3.6)	1.03 (0.93 to 1.15)
Shared use agreement^e^	43.5 (41.3 to 45.7)	37.6 (35.4 to 40.0)	–5.9 (–9.1 to –2.6)	0.87 (0.80 to 0.94)

a Centers for Disease Control and Prevention. Community-based survey of supports for healthy eating and active living (5).

b Weighted prevalence, with “don’t know” recorded as “no.”

c Unadjusted prevalence difference (absolute change) from 2014 to 2021.

d Unadjusted prevalence ratio (relative change) comparing 2021 to 2014 referent group.

e Only municipalities with schools were included (2014, n = 1,915; 2021, n = 1,768).

Prevalence increased for zoning codes related to walkable block sizes, continuous sidewalks, minimum width of sidewalks, pedestrian-friendly building orientation, and pedestrian amenities on streets. It also increased for design standards related to bicycle infrastructure during roadway expansion projects and reserving space for local jurisdictions to develop bicycle infrastructure. The largest absolute gain was for zoning codes for minimum sidewalk width, which increased from 43.9% to 52.2% — an absolute gain of 8.3 percentage points (PD = 8.3; 95% CI, 5.1–11.5). The largest relative gain was for zoning codes for block size, which increased from 14.2% to 20.9% — a relative gain of 46% (PR = 1.46; 95% CI, 1.27–1.69).

Prevalence decreased for shared-use agreements, police and security patrols in parks, and park dog leash policies. The largest absolute decline was for park patrols, which decreased from 84.6% to 75.6% — an absolute drop of 9.0 percentage points (PD = –9.0; 95% CI, –11.5 to –6.4). The largest relative decline was for shared use agreements, which decreased from 43.5% to 37.6% — a relative drop of 13% (PR = 0.87; 95% CI, 0.80–0.94) (Table 3).

### Effect modification by municipality characteristics

**Figure 1:**
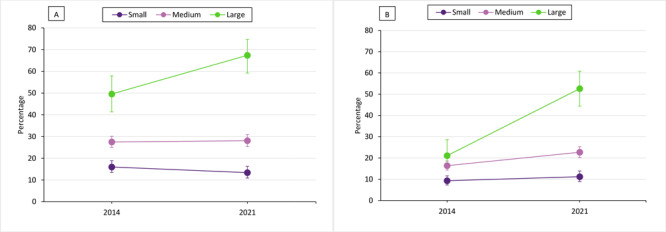
Prevalence of (A) Complete Streets Policy and (B) zoning code for block size, by population size of US municipalities. Population size is based on the 2007 and 2017 US Census of Government (15) files for respective survey administrations (large: ≥50,000 people; medium: 2,500 – 49,999 people; small: 1,000–2499 people). Source: Community-Based Survey of Supports for Healthy Eating and Active Living (CBS HEAL 2014 and 2021) (5).

Population size modified the change in prevalence of Complete Streets policies (*P* for interaction = .004) and zoning codes for block size (*P* for interaction < .001). Prevalence of Complete Streets policies remained low in small-sized municipalities (16.0% and 13.4%; *P* = .17) and in medium sized municipalities (27.5% and 28.1%; *P* = 0.75) and increased in large-sized municipalities (from 49.6% to 67.4%; *P* = .002). The prevalence gap between small and large municipalities widened from 33.6 percentage points (PP) in 2014 to 54.0 PP in 2021. A similar trend was seen for zoning codes for block size: the gap between small and large municipalities widened from 11.8 PP in 2014 to 41.4 PP in 2021 (Figure 1).

**Table:** 

A	2014, % (95% CI)	2021, % (95% CI)
Small	16.0 (13.5 –18.9)	13.4 (10.9 – 16.3)
Medium	27.5 (25.0 – 30.1)	28.1 (25.4 – 30.9)
Large	49.6 (41.4 – 57.9)	67.4 (59.2 – 74.7)
**B**		
Small	9.3 (7.3 – 11.6	11.2 (8.9 – 13.9)
Medium	16.4 (14.3 – 18.6)	22.7 (20.2 – 25.3)
Large	21.1 (15.1 – 28.6)	52.6 (44.4 – 60.8)

**Figure 2:**
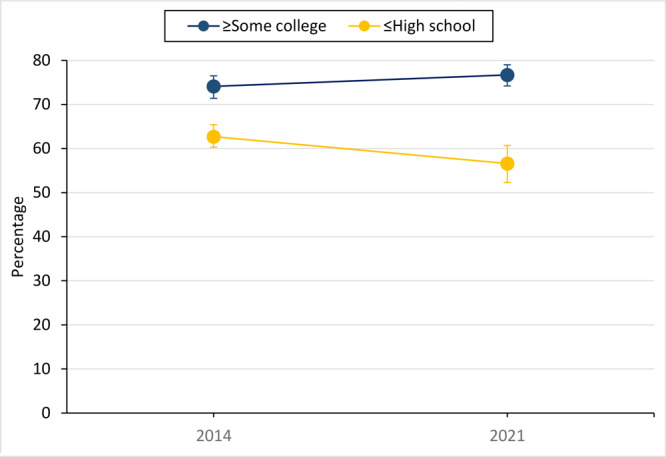
Prevalence of zoning code for mixed land use, by educational attainment of US municipalities, from the Community-Based Survey of Supports for Healthy Eating and Active Living (CBS HEAL) (5), 2014 and 2021. Educational attainment is based on the 2013 and 2020 American Community Survey (14) 5-year population estimates for respective survey administrations (some college or more, at least 50% of the population aged 25 years or older has at least some college as the highest level of formal education; high school diploma or less, at least 50% of the population aged 25 years or older has a high school diploma or less as the highest level of formal education).

Community-level educational attainment modified the change in prevalence of zoning codes for mixed land use (*P* = .007 for interaction). Municipalities in which most of the population had some college had a stable prevalence (74.1% and 76.7%; *P* = .15), whereas those where the majority had a high school diploma or less experienced a decline (from 62.7% to 56.6%; *P* = .02). This expanded the prevalence gap from 11.4 PP to 20.1 PP (Figure 2).

**Table:** 

Demographic	2014, % (95% CI)	2021, % (95% CI)
Some college or more	74.1 (71.4 – 76.5)	76.7 (74.2 – 79.0)
High school diploma or less	62.7 (60.3 – 65.4)	56.6 (52.3 – 60.7)

### Sensitivity analyses

Excluding the 424 municipalities that participated in both 2014 and 2021 did not substantially alter our findings. Of the 10 significant differences in the overall analysis, only 1 (zoning codes for pedestrian-friendly building orientation) was insignificant in the sensitivity analysis. For the 15 supports in our analysis, “don’t know” responses ranged from 2.8% for police and security patrols in parks to 21.2% for block size zoning codes in 2014 and 3.2% for parks maintenance to 19.8% for Complete Streets policies in 2021. The median was 10.3% in 2014 and 9.7% in 2021. Reassigning “don’t know” responses as missing did not substantially alter the findings.

## Discussion

In this national study of changes in physical activity–friendly policies and community design features, the prevalence of some municipal supports was higher in 2021 than in 2014. Prevalence increased for some zoning codes and design standards supportive of physical activity and decreased for shared use agreements and some parks and outdoor recreation policies. Adoption of Complete Streets policies and zoning codes for block size disproportionately increased in populous municipalities; adoption of zoning codes for mixed land use disproportionately decreased in municipalities where most of the population had less formal education compared with municipalities with populations that had some college education.

### Complete Streets

Our findings regarding Complete Streets policies illustrate a widening disparity gap. From 2014 to 2021, Complete Streets policy adoption improved from 50% to 67% in municipalities with large populations (≥50,000 residents), but not in less populous communities. In the 2014 survey, medium and large municipalities, compared with small municipalities and adjusted for geographic and socioeconomic factors, had 57% and 218% greater odds of reporting a Complete Streets policy, respectively (7). According to Smart Growth America (16), from 2014 to 2021 the number of US cities and towns with a Complete Streets policy increased from 894 to 1,520. The widening prevalence gap by population size suggests that large municipalities are recognizing the importance of polices like Complete Streets that consider the safety of all road users. It may also suggest that adopting these policies in smaller towns is less practical or unnecessary. Our results further indicate that Complete Streets policies might benefit from more publicity, because many responded “don’t know” in our questionnaire (7).

Small communities may face unique challenges in adopting Complete Streets policies, including resource limitations and limited control over key roadways (eg, where state-owned highways serve as main streets). Acknowledging these challenges, the Federal Highway Administration developed *The Small Town and Rural Multimodal Networks*, a practical resource that helps small towns and rural communities promote “safe, accessible, comfortable, and active travel for people of all ages and abilities” (17). Depending on the needs and priorities of the community, strategies to promote active transportation may include adding, expanding, or connecting sidewalks, bicycle lanes, and shared use paths; enhancing lighting, signage, and painted markings at intersections; decreasing vehicle speeds through curb extensions, roadway narrowing, and other traffic-calming measures; installing benches and climbable art for children; and landscaping along walking paths to provide a tree shade canopy (4,17–19). Given the rural–urban discrepancy in obesity and physical inactivity among children (20) and adults (21), an emphasis on improving activity opportunities in rural areas may be especially important.

### Zoning codes and design standards

Design standards that encourage bicycling and zoning codes that support walking are associated with greater physical activity for transportation (22) and for leisure (23). In the 2021 survey, prevalence increased for 7 of 9 design standards or zoning codes supportive of physical activity, and 2 supports remained consistent from 2014. Design standards that integrate bicycle infrastructure into roadway expansion and retrofit projects are important for supporting the expansion of bicycle networks. Zoning codes that widen and connect sidewalks improve safety and convenience for pedestrians. Although prevalence of these supports increased, the 2021 estimates were modest, ranging from 32% to 52%. Moreover, traffic-calming design features remained statistically flat (at 51% in 2021), despite vehicle speed being identified as the overwhelming concern for US adults who report traffic as a barrier to walking (24).

For some physical activity supports, the prevalence gap widened by municipality characteristics. The prevalence of zoning codes for mixed land use differed between municipalities with more- and less-educated populations in 2014, and this gap widened in 2021. Municipalities that serve populations with less education may have less funding and experience to support policy gaps. This finding warrants deeper investigation to identify barriers and solutions. Compared with large municipalities, medium and small municipalities (<50,000 residents) were less likely to report activity-friendly design policies, and some of these gaps also widened over time. These trends may deserve attention, because activity-friendly zoning codes have been associated with reduced economic disparities in active transportation to work (25). Our results suggest that tailoring community design approaches to promote physical activity may need to be based on municipality population size and preferences (26). Municipal leaders may consider using CDC’s Active Communities Tool to appraise the current zoning code environment, develop an action plan, and monitor progress (27).

### Parks and outdoor recreation

Parks and outdoor recreation areas contribute to the built and natural environments of physical activity–friendly communities (28). Prevalence of parks and outdoor recreation policies assessed in CBS HEAL exceeded 75% in 2021. Compared with 2014, each support had a similar or slightly reduced prevalence, with the notable exception of a 9.0 percentage point decline for police or security patrols. The Community Preventive Services Task Force recommends multicomponent interventions that support access to and use of parks, trails, and greenways. These interventions, which combine an infrastructural component (eg, playground facilities) with a non-infrastructural component (eg, community engagement efforts), are associated with greater use of parks, trails, and greenways and with expanded participation in moderate-to-vigorous leisure-time physical activity. Municipalities can apply this evidence-based strategy by ensuring adequate park lighting and signage, maintaining green space and equipment, offering outdoor recreation programs, expanding public awareness, and increasing safety (28).

### Shared use agreements

Among all supports, the largest relative decline was for shared use agreements between municipalities and schools. By permitting the public to use designated school facilities during nonschool hours, shared use agreements can expand community access to recreational facilities. This finding may be important, because inconvenience and unsafe conditions are common barriers to engaging in physical activity (6,19), although decreases may reflect temporary school closures and public health orders to minimize the impact of COVID-19. Regardless of cause, the low 2021 prevalence of shared use agreements may present an opportunity for expanding physical activity access in communities, because schoolyards may offer a convenient park space. Just as Complete Streets policies require partnership with transportation departments, municipality officials are encouraged to collaborate with school districts and the private sector to develop these agreements. A Shared Use Playbook offers practical suggestions for drafting agreements and navigating issues of funding, liability, and safety (29).

### Challenges and opportunities

In addition to up-front costs, some infrastructure changes for active living may increase property values, raising concerns about gentrification and displacement and potentially worsening physical activity disparities by income level and by race and ethnicity (30). In the 2018 SummerStyles survey, however, over half of US adults favored community development projects to make it easier to walk or bicycle, even if they increased the cost of living. Moreover, support was equally high across income and racial and ethnic groups (30). Personal safety and security — both real and perceived — are also important constructs to consider when designing activity-friendly communities (31). A recent meta-analysis found that levels of both objectively measured crime rates and subjectively measured safety concerns were associated with reduced physical activity (32).

Through funding and technical assistance, CDC is supporting communities to implement these physical activity supports. Three funding programs — State Physical Activity and Nutrition, High Obesity Program, and Racial and Ethnic Approaches to Community Health (33) — currently support 71 states and municipalities in their efforts to promote health, prevent chronic diseases, and reduce racial and ethnic health disparities. For example, High Obesity Program recipients have renovated community parks (34) and installed crosswalks and speed bumps to encourage safe walking and bicycling to everyday destinations (35). Both are strategies endorsed by CDC’s Active People, Healthy Nation initiative (36).

The increased prevalence of many zoning codes and other activity-friendly design features is encouraging, although the presence of a policy does not guarantee successful implementation. To establish intervention priorities and to operationalize policies, community members should be engaged throughout the process, from identifying their most salient needs to monitoring their implementation. Members with different concerns, access, and barriers to physical activity need to be included in these conversations. For example, the Community Preventive Services Task Force’s *Implementation Resource Guide *emphasizes the importance of disability inclusion, whereby people who use wheelchairs help identify what facilitates or hinders rolling (37). Because physical activity interventions can have broad effects on residents across age and income levels, ensuring community engagement in planning, delivering, and maintaining the intervention can also help foster civic engagement and social cohesion. Additional barriers to implementation should also be considered, including budget and training constraints and performance metrics that favor motor vehicles over other road users (38–40).

### Strengths and limitations

To the best of our knowledge, CBS HEAL is the only nationally representative survey of municipal policies that support healthy eating and active living. Nonetheless, it has some limitations, which may be reflected in the results of our study. Questions may be open to interpretation, and responses may not accurately reflect municipal code. Differences in reporting may vary according to community sociodemographic characteristics. In the 2014 survey, smaller and more rural municipalities less accurately reported Complete Streets policies, compared with the National Complete Streets Coalition’s database (7). A minor methodologic change between the 2014 and 2021 surveys could introduce another potential source of outcome misclassification bias. Principal respondents in both administrations were encouraged to contact colleagues as needed for survey completion; however, the 2021 survey streamlined that process by providing a “nominate” button on each module. Given similar frequency of “don’t know” responses in both surveys, combined with unremarkable results from the sensitivity analyses, we suspect this change did not substantially alter our findings. Survey administration in 2021, during the COVID-19 pandemic, also may have affected responses regarding shared use agreements. Finally, despite weighting the results for sampling design and nonresponse, some residual bias may exist from the lower response rate.

## Conclusion

Opportunities for physical activity can be enhanced through policy and community design interventions (3). Municipalities with these policies are situated to cultivate activity-friendly environments, the benefits of which may extend beyond personal health to a stronger local economy, cleaner air quality, and greater community development (4). Findings from CBS HEAL 2021 showed that prevalence of many municipal supports for physical activity was higher in 2021 than in 2014. Nonetheless, disparities by population size and education level widened for Complete Streets policies and some zoning codes supportive of physical activity. US municipalities can consider adopting activity-friendly zones, parks and outdoor recreation policies and budgets, design standards, Complete Streets policies, and shared use agreements to improve access to safe environments for physical activity for people of all ages and abilities.
